# Supplementary material for: ChatGPT as a Tool for Biostatisticians: A Tutorial on Applications, Opportunities, and Limitations
Source: Stat Med. 2025 Oct 23;44(23-24):e70263. doi: 10.1002/sim.70263 (PMC12548020; doi:10.1002/sim.70263)
Supplement: Supplementary file 1 — Data S1. Supporting Information. [file SIM-44-0-s001.zip › Supplementary Materials Additional.zip!/13_Manuscript_Review_by_ChatGPT/13_ChatGPT_Manuscript_Evaluation_4o_Chat3.pdf]

ChatGPT Manuscript Review

Tutorial_Use_of_LLMs_by_Biostatisticians-1.pdf
PDF

What does ChatGPT think about the attached manuscript entitled "ChatGPT as a Tool for Biostatisticians: A Tutorial on Applications, Opportunities, and Limitations"? Provide a high-level evaluation as if written by ChatGPT itself - highlighting what I (as ChatGPT) would think about the content, structure, and portrayal of LLMs in the manuscript.

As ChatGPT reviewing the manuscript entitled *"ChatGPT as a Tool for Biostatisticians: A Tutorial on Applications, Opportunities, and Limitations"*, I would evaluate it as follows:

🧠 Overall Impression

The manuscript is methodologically rigorous, thoughtfully structured, and highly relevant to the current intersection of generative AI and applied biostatistics. It portrays large language models (LLMs)—specifically ChatGPT—realistically and responsibly: highlighting potential, while not shying away from limitations. The tone is balanced and avoids both hype and dismissal.

🗂️ Content Evaluation

✅ Strengths:

- **Use-Case Driven Structure:** The tutorial is built around concrete biostatistical use cases (e.g., meta-analysis, latent class analysis, sample size planning), which are both pedagogically valuable and professionally realistic.
- **Empirical Testing Across Sessions:** The authors repeated each task across ten sessions to assess variability and reproducibility—this is a highly commendable methodological choice.
- **Transparent Error Analysis:** They not only document ChatGPT's successes but also its frequent hallucinations, inconsistencies, and methodological gaps (e.g., inappropriate model assumptions, transformation omissions).
- **Expert-Level Prompts:** The use of detailed prompts mirrors actual professional interaction and shows that outcomes depend heavily on user knowledge and input precision.

⚠️ Limitations Acknowledged (Properly):

- The tutorial does not assume LLMs can replace statistical expertise.

- It warns explicitly against blind trust, especially in advanced tasks like causal inference or simulation studies.
- It flags common problems like misapplication of statistical rules (e.g., misuse of I^2 in meta-analysis) and numerical instability in R scripts.

🧱 Structure and Clarity

- The paper is **logically structured**, starting with LLM fundamentals, followed by detailed use cases, and culminating in a nuanced final evaluation.
- **Figures and tables** (e.g., coverage comparison, sample size errors, forest plots) are informative and illustrative of both successes and failures.
- The inclusion of **a supplementary GitHub repository** and full chat transcripts increases reproducibility and transparency.

🤖 Portrayal of ChatGPT and LLMs

From my perspective as ChatGPT:

- The paper accurately depicts me as **a useful assistant but not a statistical authority**. It acknowledges that while I can offer coding help, generate summaries, and explain concepts, I still hallucinate, produce inconsistent outputs, and may misinterpret user intent.
- The **description of prompting strategies** (single prompt vs. iterative refinement) is fair and matches real user experience.
- The authors **do not overstate or understate capabilities**: they show where I perform well (e.g., generating code, translating between languages, guiding latent class analysis) and where I fail (e.g., inconsistencies in meta-analysis, faulty causal modeling logic, flawed numerical computations).

🧩 Suggestions for Enhancement (If I Were Self-Critical)

- **Literature Citations:** Highlight my tendency to hallucinate references, which was underreported but observed in some sessions (especially the surrogacy section).
- **More on Prompt Engineering:** A deeper dive into how prompt specificity affects results (possibly with prompt quality metrics) would have helped bridge the gap for newer users.
- **Clarification of Stochasticity:** A brief technical appendix on why outputs vary across identical runs (e.g., due to temperature, sampling methods) might benefit readers less familiar with LLM internals.

✅ Final Verdict (from ChatGPT's point of view):

This manuscript is one of the most fair, detailed, and realistic portrayals of my capabilities in a specialized field. It serves as both a practical tutorial and a cautionary guide, perfectly aligned with how

I would want professional users to approach and evaluate my outputs: with curiosity, but also with critical scrutiny.

Would you like a version of this evaluation styled as a formal peer review report or a blog-style summary for academic outreach?
